# Effect of Different Austempering Heat Treatments on Corrosion Properties of High Silicon Steel

**DOI:** 10.3390/ma14020288

**Published:** 2021-01-08

**Authors:** Mattia Franceschi, Luca Pezzato, Alessio Giorgio Settimi, Claudio Gennari, Mirko Pigato, Marina Polyakova, Dmitry Konstantinov, Katya Brunelli, Manuele Dabalà

**Affiliations:** 1 Department of Industrial Engineering, University of Padua, Via Marzolo 9, 35131 Padova, Italy; mattia.franceschi@phd.unipd.it (M.F.); alessiogiorgio.settimi@unipd.it (A.G.S.); claudio.gennari@unipd.it (C.G.); mirko.pigato@unipd.it (M.P.); katya.brunelli@unipd.it (K.B.); manuele.dabala@unipd.it (M.D.); 2 Department of Mechanical Engineering and Metallurgical Technologies, Nosov Magnitogorsk State Technical University, pr. Lenina, 38, 455000 Magnitogorsk, Russia; m.polyakova-64@mail.ru (M.P.); const_dimon@mail.ru (D.K.)

**Keywords:** high-silicon steel, austempering, retained austenite, corrosion resistance

## Abstract

A novel high silicon austempered (AHS) steel has been studied in this work. The effect of different austenitizing temperatures, in full austenitic and biphasic regime, on the final microstructure was investigated. Specimens were austenitized at 780 °C, 830 °C, 850 °C and 900 °C for 30 min and held isothermally at 350 °C for 30 min. A second heat treatment route was performed which consisted of austenitizing at 900 °C for 30 min and austempering at 300 °C, 350 °C and 400 °C for 30 min. Scanning electron microscopy (SEM) and X-ray diffraction (XRD) have been used to evaluate the microstructural evolution. These techniques revealed that the microstructures were composed of carbide-free bainite, ferrite, martensite and retained austenite (RA) in different volume fractions (Vγ). An aqueous borate buffer solution with 0.3 M H_3_BO_3_ and 0.075 M Na_2_B_4_O_7_∂10H_2_O (pH = 8.4) was used for corrosion tests in order to evaluate the influence of the different volume fractions of retained austenite on the corrosion properties of the specimens. The results showed that when increasing the austenitization temperatures, the volume fractions of retained austenite reached a maximum value at 850 °C, and decrease at higher temperatures. The corrosion properties were investigated after 30 min and 24 h immersion by means of potentiodynamic polarization (after 30 min) and electrochemical impedance spectroscopy (after both 30 min and 24 h) tests. The corrosion resistance of the samples increased with increases in the volume fraction of retained austenite due to lower amounts of residual stresses.

## 1. Introduction

In the latest years, significant interest has been dedicated to austempered high-silicon steels (AHS) [1,2]. High strength, toughness and ductility characterize these steel grades, making them very attractive materials. Their technological application can be in several industrial fields, such as automotive, mining, machinery, etc. [3].

Silicon is the most important element for these alloys, and it determines the final microstructural constituents and their amount [4]. It inhibits carbides precipitation, in particular cementite [5]. The suppression of cementite precipitation allows the partitioning of carbon into austenite during bainitic transformation, increasing its carbon content. This increases the stability of austenite, favoring its retention at room temperature.

Zhu et al. [6] observed the strong silicon contribution to the recrystallization processes; silicon delayed dynamic recrystallization and FCC iron grain growth, further favoring austenite retention.

In order to obtain the best microstructure, these steels have to be subjected to a particular heat treatment (HT) consisting of several steps. A heating phase in the dual phase, austenitic–ferritic, or in the austenitic region, represents the first step of the treatment. Austenitizing in a dual phase regime should be preferable because it contributes to carbon partitioning and retained austenite (RA) stabilization. In fact, newly formed ferrite rejects carbon that diffuses into austenite, which becomes more stable. After the austenitizing step, the material is cooled to the austempering temperature (AT) that is between bainite start (Bs) and martensite start (Ms). Once the selected AT is reached, the material is isothermally held and then cooled to room temperature [4,7].

The main purpose of this HT is to obtain a microstructure consisting of RA and free carbide bainite, which exhibits a high strength and is tougher than martensite [8]. In several cases, it is preferable to avoid the presence of RA, because it leads to a strong reduction in the mechanical properties, as reported by Pezzato et al. [9]. However, in modern AHSS-retained austenite has great importance due to its capability to undergo martensitic transformation when subjected to mechanical stresses. This phenomenon is called the transformation-induced plasticity (TRIP) effect, and enhances mechanical properties (i.e., strength, fracture strain, uniform strain, work hardening rate, etc.) [3,4,10,11].

The second aim of the treatment is precisely the stabilization of austenite at room temperature. Austenite’s stability is influenced by several factors, such as carbon content, grain size, orientation and residual stresses [12,13,14]. Austenite’s stability increases with its carbon content, reducing the grains size; moreover, non-preferred orientation and the absence of textures favor its retention.

RA is characterized by two morphologies: (a) film-like austenite, and (b) blocky austenite [15]. Filmy austenite is located between bainitic ferrite laths, and its presence is attributed to silicon, which prevents the precipitation of cementite. Supersaturated ferrite rejects the carbon that enriches austenite, which becomes stable at room temperature. Films of austenite have enhanced strength, toughness and wear resistance due to their low tendency to exhibit the TRIP effect, and their being an obstacle for cracks propagation [4,15,16].

The carbon content in blocky austenite is lower than in film-like RA and is inhomogeneous. These characteristics make RA more susceptible to martensitic transformation, and it transforms at lower deformation levels in comparison with filmy austenite. The carbon inhomogeneity inside RA blocks provokes a gradual transformation: the poorest zones, within the grain, transform before the richer ones. Austenite blocks also have lower mechanical stability because they contain a large number of nucleation sites for martensite formation and less constraints for the transformation [15,16].

The microstructure and the final mechanical properties of steel depend on the heat treatment parameters [17]. Concerning the HT parameters effects on microstructure, in the literature there are numerous works and results focused on the soaking step at the AT temperature. Palaksha et al. [18] observed that when increasing the austempering temperature from 275 to 350 °C, the final volume fraction of retained austenite increases, then a further temperature increase leads to a decrease in RA stabilization. Such an effect has been observed also by Putatunda [2], who demonstrated that the same trend can be observed for carbon content in RA and material fracture toughness. Son et al. [19] observed on a 0.9C-2.3Si steel an increase in both ferritic and austenitic grain size, and RA final amount. Kumar et al. [20] demonstrated that the bainite volume fraction increases with a decreasing austempering temperature, and that RA decreases with holding time. The reduction in the final volume fraction of the retained austenite’s increasing isothermal bainitic transformation (IBT) time was also observed by Acharya et al. [21]. Concerning the effect of austenitizing temperature, Zhao et al. observed that increasing austenitizing temperatures led to coarser prior austenite grain size (PAGB) and bainitic packets, coarser RA blocks, and a higher final volume fraction of the retained austenite [22].

The authors in a previous work [17] reported results for the effect of different austenitizing conditions; as the austenitization temperature increased, a grain coarsening was observed. Moreover, this led to the presence of bainitic ferrite (BF), promoted by element diffusion [23].

Microstructural modification induced by heat treatments could influence corrosion resistance, as observed by several authors both in low-alloyed carbon steels [24] and in high-alloyed steel, such as stainless steel [25] and duplex stainless steel (DSS) [26].

Concerning the effect of retained austenite on corrosion resistance, in the literature there are several studies. Bignozzi et al. [27], studying a high-chromium martensitic steel, observed that when increasing the volume fraction of RA, the corrosion rate decreased due to the low amount of internal stress in the retained austenite [28,29]. Additionally, Han et al. [30] observed that when increasing the volume fraction of austenite and the nodularity of graphite in cast iron, the corrosion rate decreased.

In this work, the effects of different austenitizing temperatures at fixed AT and the effects of different ATs at the fixed austenitizing temperature were investigated. Microstructural evolution was evaluated with electron microscopy and X-ray diffraction.

In addition, given the lack of data in the literature regarding the corrosion resistance of these steel grades, the effect of the microstructural constituents and RA content on the corrosion resistance of the material was investigated in borate buffer solution [31].

## 2. Materials and Methods

High silicon steel was supplied by Nosov Magnitogorsk State Technical University (NMSTU) in the form of 8 mm diameter wires. Chemical composition, given in weight percentage (wt.%), is summarized in Table 1.

Carbolite tubular and Nabetherm 3000 electric furnaces were used for the realization of austempering heat treatments. The first was used for the normalizing treatment (900 °C and calm air-cooling) and for the austenitizing step. The second was used for the austempering step, i.e., soaking at AT for isothermal bainitic transformation (IBT). An air-cooling system was used for the cooling step from the austenitization temperature to the AT temperature, and a water-cooling system for the final cooling to room temperature.

**Figure 1:**
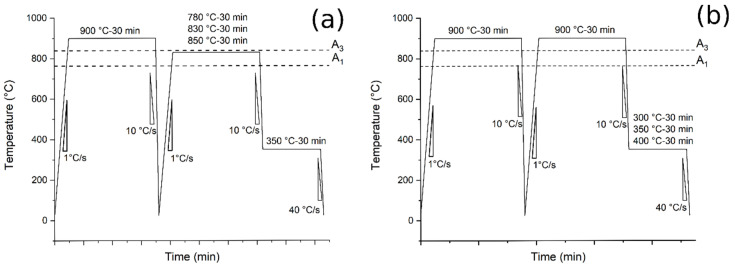
Heat treatments scheme: (**a**) the tests performed at different austenitizing and fixed austempering temperatures (route 1); (**b**) the tests performed at fixed austenitizing and different austempering temperatures (route 2).

The subsequent treatments were performed (summarized in Table 2):

- *Normalizing:* heating at 900 °C at 1 °C/s, 30 min holding time and air-cooling (10 °C/s);
- *Austempering (Route 1)*: Pre-normalization treatment from 900 °C (30 min) and water-cooling. Heating at 780, 830, 850 °C for 30 min at 1 °C/s, air cooling at 10 °C/s to 350 °C and holding for 30 min followed by water-cooling to room temperature at 40 °C/s (Figure 1a);
- *Austempering (Route 2)*: pre-normalization treatment from 900 °C (30 min) and water-cooling. Heating at 900 °C for 30 min at 1 °C/s, air cooling at 10 °C/s to 300, 350 and 400 °C and holding for 30 min followed by water-cooling to room temperature at 40 °C/s (Figure 1b).

Microstructure evolution was investigated with a scanning electron microscope (SEM, Leica Cambridge Leo Stereoscan 440, Leica Microsystems S.r.l., Milan, Italy), operating with a secondary electron (SE) at 15 kV and in backscattered electron mode (BSE) at 25 kV. Heat-treated specimens were cut along the cross section with lubricated SiC disks, mounted using phenolic resin, ground with abrasive SiC papers (from 320 to 1200 grit) and polished using clothes and polycrystalline diamond suspensions of 6, 3 and 1 µm. In order to reveal microstructures, Nital 2 (2 mL of nitric acid in 98 mL of ethanol) etchant was used.

Phase identification and quantification were performed with X-ray diffraction, using a Siemens D500 X-ray diffractometer (Siemens, Munich, Germany), equipped with a monochromator on the detector side and a Cu radiation tube, working at 40 kV and 30 mA. The 2θ = 40–105° angular range was investigated with a step scan of 0.025° and a counting time of 6 s per step. The evaluation of the volume fraction of the phases was performed by means of Rietveld analysis with Maud software (Lutterotti, University of Trento, Department of Material Engineering, Trento, Italy).

The corrosion performances were investigated by potentiodynamic polarization tests (PDP) at room temperature. Polarization tests were performed in a borate buffer solution containing 0.3 M H_3_BO_3_ and 0.075 M Na_2_B_4_O_7_•10H_2_O (pH = 8.4) [31], using an AMEL 2549 Potentiostat (Amel Electrochemistry S.r.l., Milan, Italy). The reference electrode was a saturated calomel electrode (SCE), the counter electrode was a platinum electrode, and the scan rate was 0.5 mVs^−1^ in a potential range between OCP−0.2V (0.2 V below the measured OCP) and +2.5 V. The electrolyte was chosen in order to simulate a non-strongly aggressive environment to underline the effect of retained austenite in the material. Corrosion data (*E_corr_* and *I_corr_*) were extrapolated graphically using the Tafel method.

EIS (electrochemical impedance spectroscopy) tests were also performed using the same cell and electrolyte of the PDP at the value of the open circuit potential, and in a range of frequency 10^5^–10^−2^ Hz with a perturbation amplitude of 10 mV, after 30 min and 24 h of immersion. An AMEL 2549 Potentiostat (Amel Electrochemistry S.r.l.) coupled with a Materials Instrument Spectrometer was employed. The fitting of experimental data was carried out using the software ZView (Scribner Associates Inc, Southern Pines, NC, USA) and the Randles circuit as the equivalent circuit. Both PDP and EIS tests were performed after 30 min of OCP stabilization. Each measure was repeated three times in order to assure statistical reproducibility.

## 3. Results and Discussion

### 3.1. Microstructural Characterization

The microstructure of the samples was analyzed along the cross section by electron microscopy.

**Figure 2:**
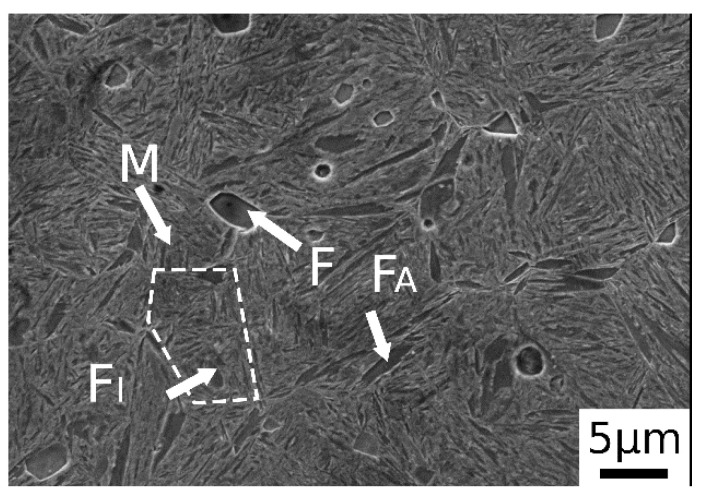
SE-SEM image of material after normalization (M martensite, F ferrite, F_A_ allotriomorphic ferrite and F_I_ idiomorphic ferrite).

After normalization, as shown in Figure 2, the material exhibited a microstructure consisting of martensite and ferritic islands, located at the grain boundaries (allotriomorphic ferrite, F_A_) and in the center (idiomorphic ferrite, F_I_). The presence of martensite was due to the high amounts of alloying elements, which switched the transformation curves to higher times, decreasing the critical cooling rate necessary to avoid ferrite and pearlite formation. The trace of the prior austenite grain boundary is indicated by the dashed line in the figure.

**Figure 3:**
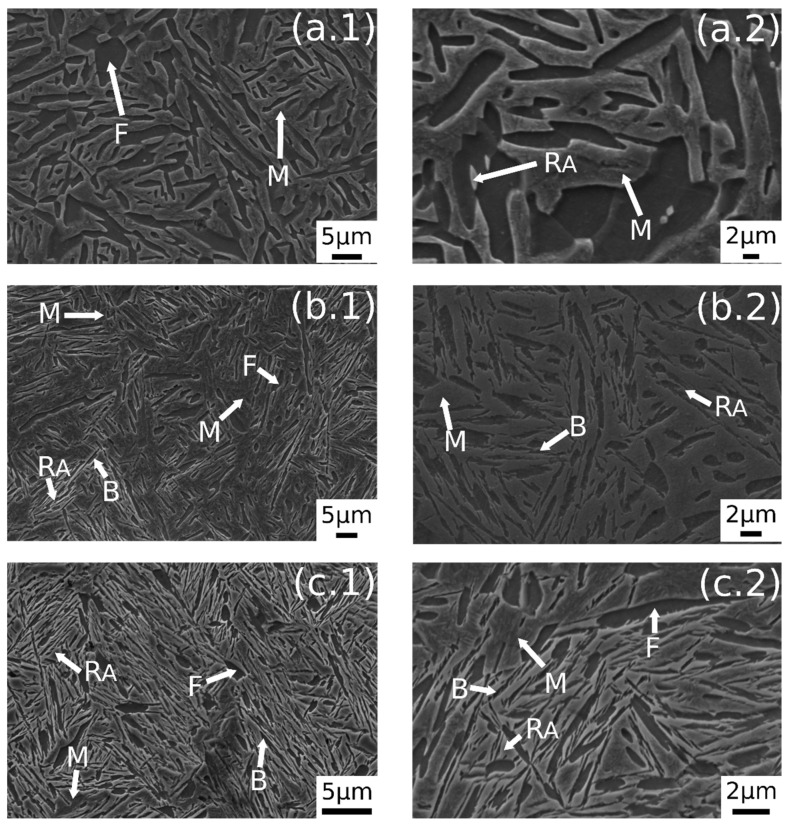
SE-SEM images of the surfaces of the samples: (**a.1**,**a.2**) 780 °C + 350 °C, (**b.1**,**b.2**) 830 °C + 350 °C, (**c.1**,**c.2**) 850 °C + 350 °C. (M martensite, F ferrite, R_A_ retained austenite, B carbide free bainite).

In Figure 3 are reported the SEM images after austempering performed at different austenitizing temperatures. In Figure 3a.1,a.2 it is possible to observe a dual-phase microstructure consisting of different ferrite morphologies and martensite. A similar microstructure was observed also by the authors in a previous research [23]. The austempering treatment in this condition does not lead to bainite formation. As state in ref. [23], this phenomenon could be explained by the carbon partitioning that changes Bs temperature, or due to the short soaking time at AT.

**Figure 4:**
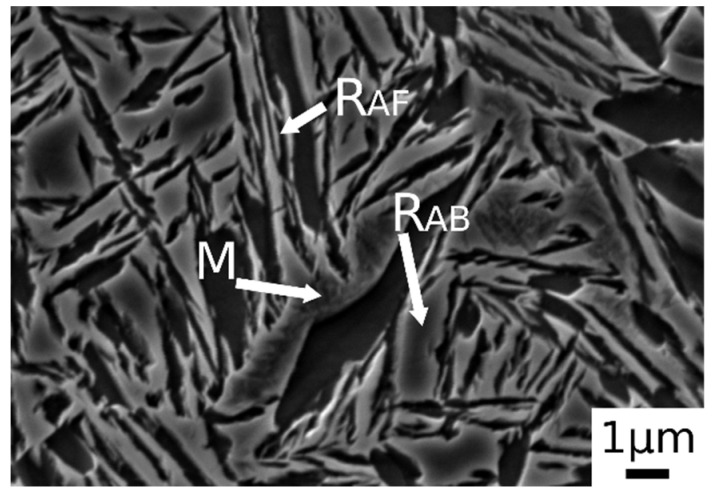
SE-SEM image at high magnification of the alloy after austenitizing at 850 °C (30 min) and austempering at 350 °C for 30 min. (M martensite, R_AB_ blocky retained austenite, R_AF_ filmy retained austenite).

Figure 3b.1,b.2 shows the microstructure after austenitizing for 30 min at 830 °C. In this case the presence of carbide free bainite (B) can be noted. The microstructure consisted of a martensite matrix with ferritic islands and free-carbide bainite sheaves [16]. Increasing the austenitizing temperature to 850 °C (Figure 3c.1,c.2), the same microstructure is observed. The coarsening of bainitic sheaves should be observed when increasing the temperature from 830–850 °C, according to the literature [32], together with a decreasing width of martensitic regions. Figure 4 shows a high magnification image of the specimen treated at 850 °C for 30 min and austempered at 350 °C. It is possible to distinguish, as reported by numerous authors in the literature [15,16,33], two morphologies of retained austenite: (a) blocky type RA (R_AB_), with a low carbon concentration and a high tendency to exhibit a TRIP effect; (b) high-carbon film shape austenite (R_AF_) with high mechanical stability. Laths of martensite are also visible (M).

**Figure 5:**
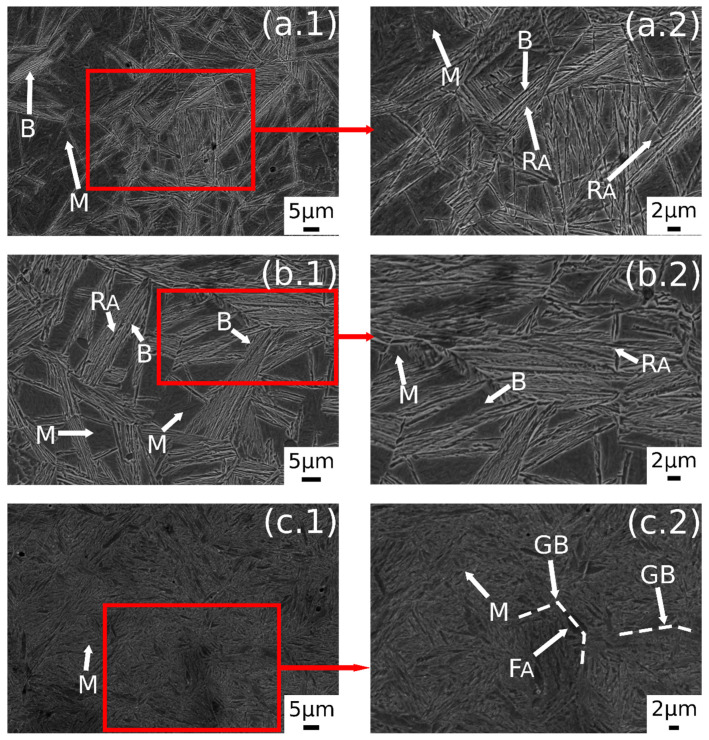
SE-SEM images of the surfaces of the samples: (**a.1**,**a.2**) 900 °C + 300 °C, (**b.1**,**b.2**) 900 °C + 350 °C, (**c.1**,**c.2**) 900 °C + 400 °C. (M martensite, F ferrite, R_A_ retained austenite, B carbide free bainite, GB prior austenite grain boundary, F_A_ allotriomorphic ferrite).

A further increase in austenitizing temperature (900 °C), maintaining 350 °C of austempering temperature, led to carbide-free bainitic sheaves coarsening, as can be noted in Figure 5b.1,b.2. The microstructure consisted of a martensitic matrix, as before, but with a higher amount of bainite.

A prevalent martensitic microstructure with free-carbide bainite sheaves randomly oriented was observed in the sample austenitized at 900 °C for 30 min and soaked at 300 °C for 30 min, as shown in Figure 5a.1,a.2. A lower amount of bainite was found with decreasing the AT temperature. This microstructural evolution could be related to the effect of AT on the kinetic of bainitic transformation. In fact, higher austempering temperatures fasten the bainitic transformation kinetics [4]. The observed microstructural evolution agreed with the literature [1,2,34,35].

**Figure 6:**
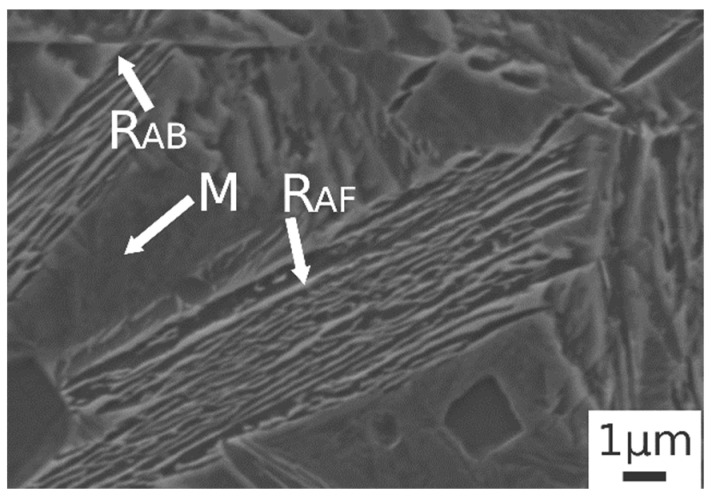
SE-SEM image at high magnification of the alloy after austenitizing at 900 °C (30 min) and austempering at 350 °C for 30 min. (M martensite, R_AB_ blocky retained austenite, R_AF_ filmy retained austenite).

Austempering at 400 °C produced a microstructure consisting of martensite (Figure 5c.1,c.2) without bainite, due to austempering at a temperature superior to Bs, as demonstrated in [23]. In the image are also indicated the trace of prior austenite grain boundaries (GB) with allotriomorphic ferrite (F_A_) [22]. R_AB_ and R_AF_ are recognizable also in the sample subjected to 900 °C austenitizing and 350 °C austempering (Figure 6). High-magnification micrographs allow for demonstrating that filmy austenite (R_AF_) is located between the bainitic laths, while the R_AB_ are located at bainitic region boundaries.

### 3.2. X-ray Diffraction

**Figure 7:**
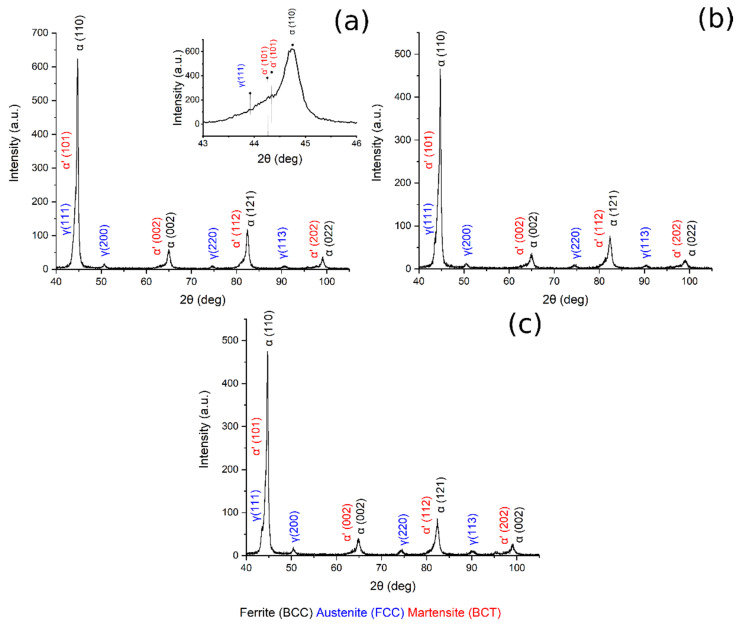
XRD pattern (**a**) 780 °C + 350 °C, in the box upon on the right can be found a zoom of the pattern in the range 43–46°, (**b**) 830 °C + 350 °C, (**c**) 850 °C + 350 °C.

X-ray diffraction patterns of austenitized samples at 780 °C, 830 °C, 850 °C are shown in Figure 7. In all the patterns are visible BCC iron peaks related to ferrite and bainite, body-centered tetragonal iron peaks related to martensite, and FCC iron peaks related to the presence of retained austenite. Displacement between ferrite and martensite peaks should explained by the high martensite carbon content and strong cell distortion [23,36].

**Figure 8:**
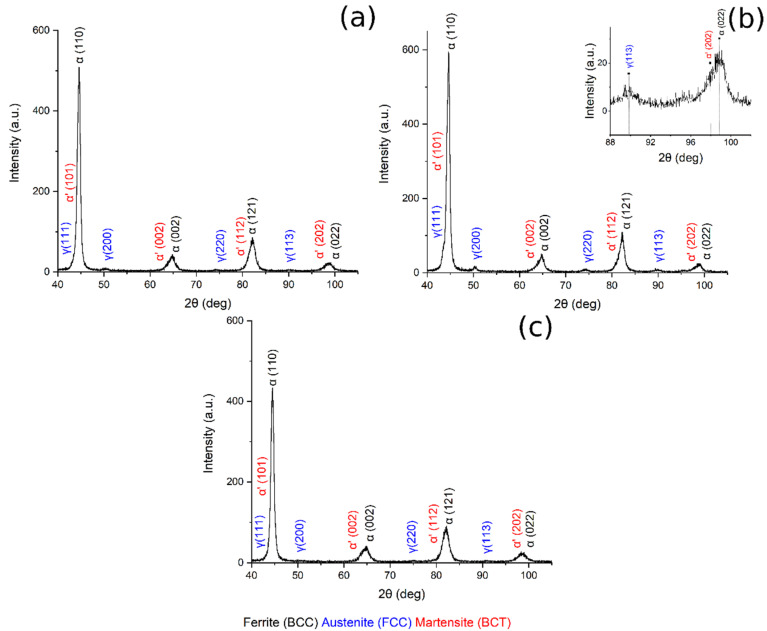
XRD pattern (**a**) 900 °C + 300 °C, (**b**) 900 °C + 350 °C, in the box upon on the right can be found a zoom of the pattern in the range 88–102°, (**c**) 900 °C + 400 °C.

Figure 8 shows the XRD pattern of the sample austenitized at 900 °C and soaked at 300, 350 and 400 °C for 30 min.

Table 3 reports the volume fraction of the phases calculated by means of the Rietveld analysis performed with the Maud software on the XRD patterns (Figure 7 and Figure 8). The results show that the microstructure mainly consisted of martensite and carbide free bainite with RA.

**Figure 9:**
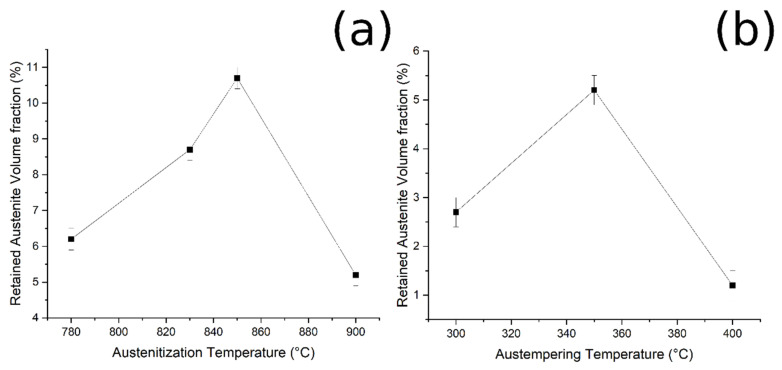
Relationship between (**a**) RA and austenitizing temperature, and (**b**) RA and austempering temperature (fixed austenitizing at 900 °C).

By plotting the austenite volume fraction vs. the austenitizing temperature, it can be observed that (i) increasing the austenitizing temperature, from 780 °C to 850 °C, produced an increase in final Vγ, and that (ii) above 850 °C a decrease in the Vγ can be noted (Figure 9a).

Furthermore, when the material was austenitized at 900 °C, the volume fraction of retained austenite increased from 300 °C to 350 °C of austempering, and then decreased (Figure 9b). These results could be explained by considering the bainitic transformation process and the kinetics: the higher the carbide-free bainite volume fraction, the higher is the amount of RA stabilized and retained at room temperature. The obtained results agree with the literature [2,17,27].

### 3.3. Corrosion Resistance

**Figure 10:**
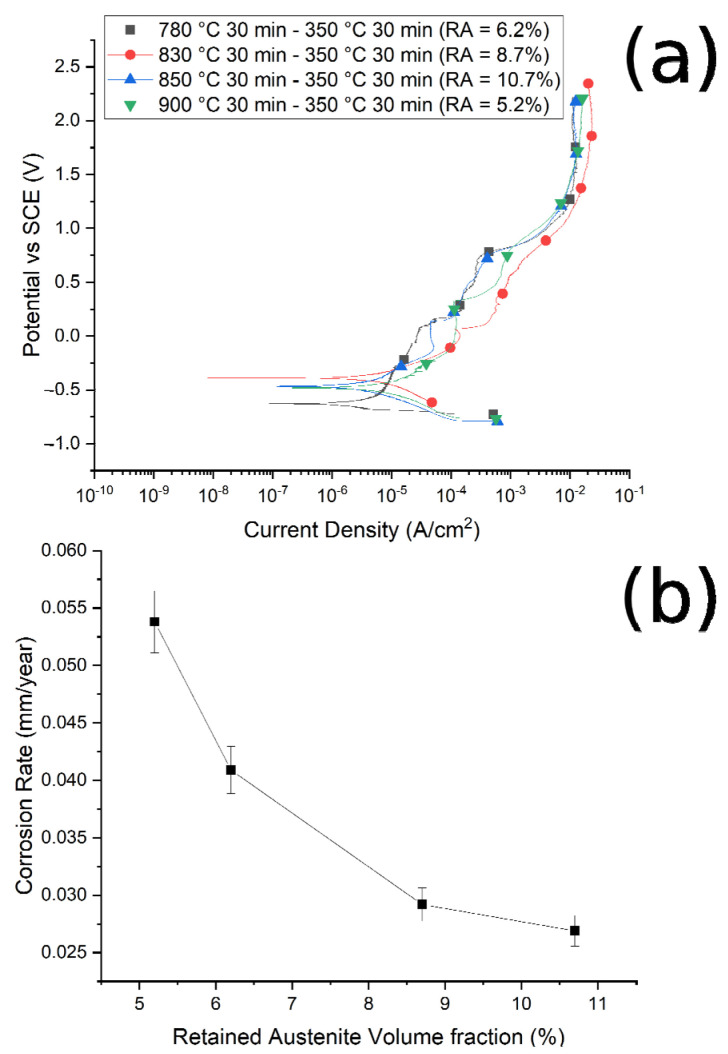
(**a**) Potentiodynamic polarization curves for the different samples austenitized at 780 °C, 830 °C, 850 °C, 900 °C and austempered at 350 °C. (**b**) Correlation between corrosion rate and RA volume fraction. Test performed in borate buffer solution.

**Figure 11:**
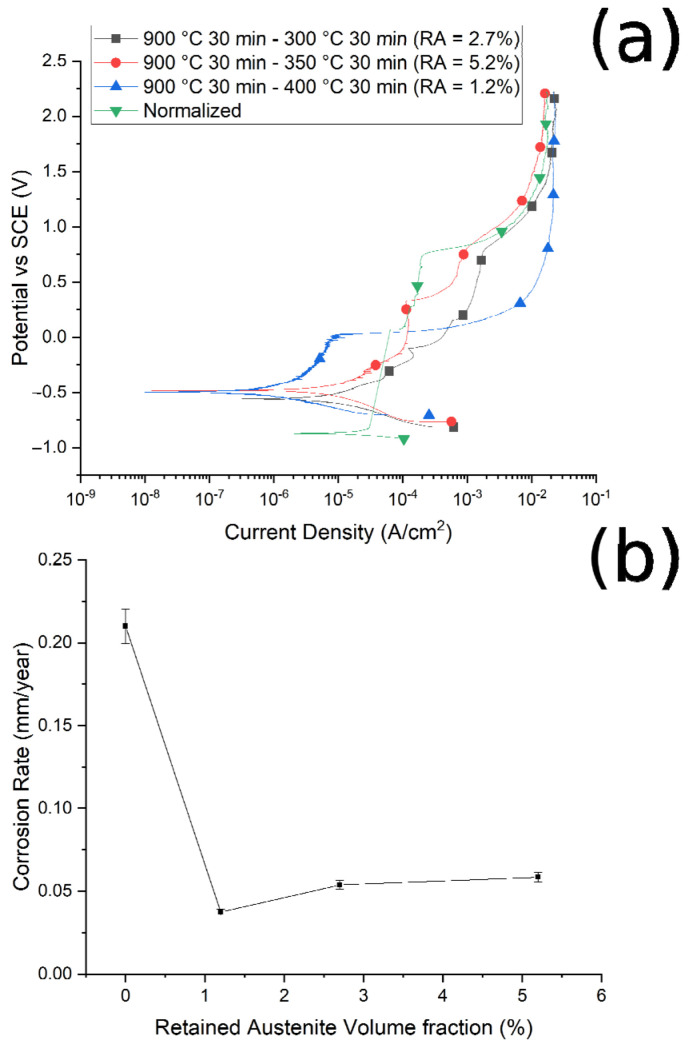
(**a**) Polarization curves of the samples austenitized at 900 °C and austempered at 300 °C, 350 °C, and 400 °C, and normalized at 900 °C. (**b**) Relationship between corrosion rate and volume fraction of retained austenite. Test performed in borate buffer solution.

The corrosion resistance of the specimens was preliminarily measured by PDP tests, in a borate buffer solution, and the PDP curves are reported in Figure 10a and Figure 11a. Values of corrosion potentials and current densities, graphically extrapolated from the curves, together with the corrosion rate, calculated with Equation (1), are reported in Table 4.

It is possible to note that, as the austenitizing temperature and volume fraction of the retained austenite increased, the *I_corr_* decreased, the corrosion rate (CR) decreased and the corrosion resistance increased, in agreement with Han et al. [30]. In Figure 10b and Figure 11b are reported the trend of the corrosion rate (CT) with the RA volume fraction for the two studied routes. A clear correlation between the quantity of RA and the CR can be noted, especially in the case of route 1, with a decrease in the corrosion rate with the increase in the RA volume fraction. This phenomenon agrees with the results of Bignozzi et al. [27], who studied the effect of austenite on martensitic stainless steel’s resistance. The effect of RA was explained by Hill et al. [28,37]: austenite was characterized by a lower amount of internal stress with respect to martensite. According to the authors, higher stresses led to a corrosion rate increase.

The results were confirmed by Hsu et al. [38], who studied the corrosion behavior of ADI cast iron in a 3.5% NaCl environment. According to their results, retained austenite, produced by austempering, acts as a corrosion inhibitor, lowering the material degradation with high efficiency.

Moreover, the results were confirmed in the research conducted by Yang et al. [39]. The authors in ref. [38] analyzed the difference in corrosion resistance between the quenching and tempering treatment (Q&T) and the quenching and partitioning (Q&P) of a medium carbon steel, in a sodium chloride solution. The investigated alloy, after Q&P, was characterized by a high amount of RA (~17.5%) with respect to the sample treated by Q&T, and this exhibited better corrosion properties both in short- and long-time immersion.

It can be noted that, at fixed austenitizing temperatures (900 °C), when increasing the austempering temperature, specimens showed better corrosion resistance, in agreement with the literature [40]. For this austenitizing temperature, the variations in the corrosion properties are smaller than in the case of route 1, as can be observed when comparing Figure 11b with Figure 10b, due to the lower variation in RA content between the various samples.

Considering the corrosion potentials (*E_corr_*), an ennoblement in the corrosion potential can be observed for all the treated samples in comparison to the normalized sample (with no RA), confirming the increased corrosion performances of the samples that contained RA. In fact, an increase in the corrosion potential indicates and ennoblement of the tested material, and so an increase in the corrosion properties.

**Figure 12:**
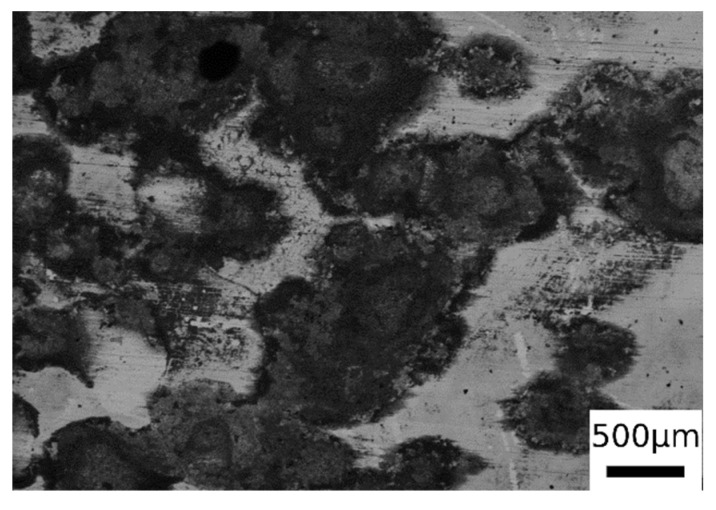
SEM micrographs surface of one sample after PDP tests (BSE mode) for the sample austenitized at 900 °C (30 min) and austempering at 350 °C for 30 min.

From the observation of the surface of the corroded samples (in Figure 12 is presented an example, but all the samples present the same surface after corrosion), it was possible to recognize mainly a uniform corrosion, with some localized corrosion sites, in accordance with the employed electrolyte. This allowed the application of the formula for corrosion rate (1) calculation reported in ASTM G102 [41],

(1) $$CR=K_{i}\cdot \frac{I_{corr}}{\rho }EW$$

where:

(a) *CR* is the corrosion rate (mm/year);
(b) *K_i_* is a constant equal to 3.27 × 10^−3^;
(c) *I_corr_* is the current density (µA/cm^2^);
(d) *ρ* is the material density, assumed equal to 7.8 g/cm^3^;
(e) *EW* is the so-called equivalent weight, calculated according to (2),

(2) $$EW=\frac{1}{\sum_{i=1}^{n}\frac{n_{i\cdot f_{i}}}{W_{i}}}$$

and *n_i_*, *f_i_* and *W_i_* represent respectively the valence, the mass fraction and the atomic weight of the *i*th element.

Further investigation on the corrosion behavior was performed with EIS tests on the samples with the best and the worst corrosion resistance, after 30 min and 24 h immersion in borate buffer solution.

**Figure 13:**
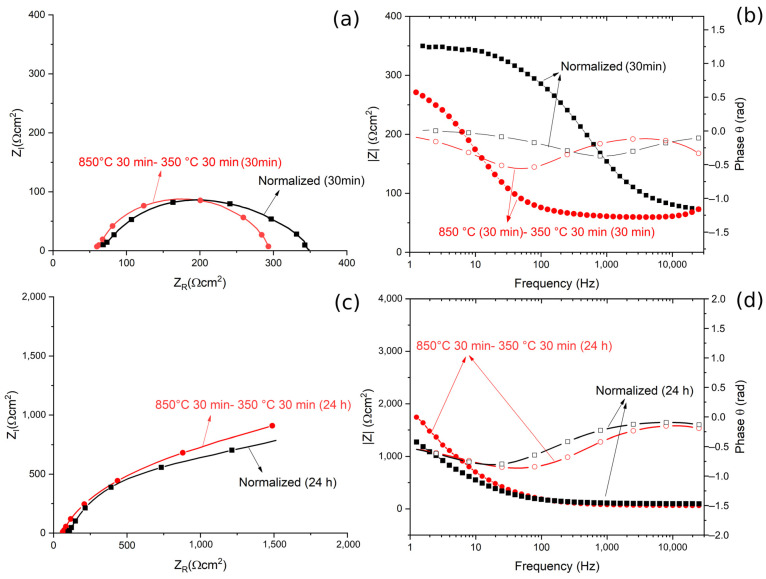
(**a**) Nyquist plot for the material austempered at 350 °C after austenitizing at 850 °C and in normalized conditions for 30 min immersion. (**b**) Bode modulus and phase plot for or the material austempered at 350 °C after austenitizing at 850 °C and in the normalized condition for 30 min immersion. (**c**) Nyquist plot for the material austempered at 350 °C after austenitizing at 850 °C, and in normalized condition for 24 h immersion. (**d**) Bode and phase plots for the material austempered at 350 °C after austenitizing at 850 °C and in normalized condition for 24 h immersion. All the tests were performed in borate buffer solution. Dots represent experimental data, lines the results of the fitting.

The results, in term of Nyquist, Bode Modulus and Phase diagrams, are reported in Figure 13.

**Figure 14:**
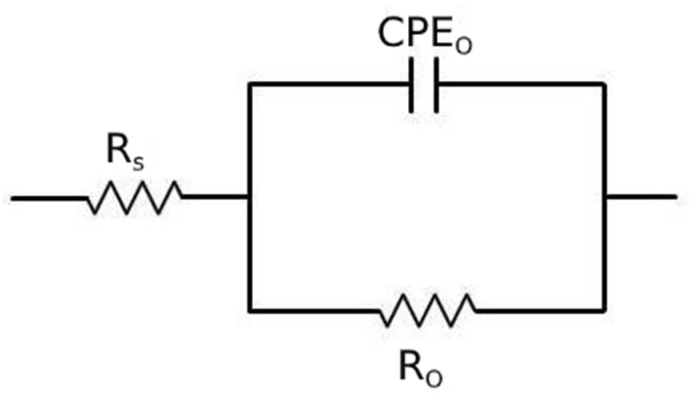
Equivalent circuit used for the fitting of EIS data.

The obtained EIS data were fitted using the equivalent circuit shown in Figure 14, called Randles circuit, and the results are reported in Table 5. In this circuit, R_S_ represents the resistance of the electrolyte, R_0_ is the polarization resistance, which depends on the passive oxide film and is a measure of the corrosion resistance of the material, and CPE_O_ is a constant phase element, used instead of a capacitance because the measured capacitance is not ideal. The choice of the equivalent circuit is in accordance with the shape of the Bode plots, which showed a single time constant, and with the literature regarding natural oxide films on steels [42].

The quality of the fitting of EIS data after both 30 min and 24 h of immersion was good, as confirmed by the low values of chi squared.

From the Nyquist plots a qualitative evaluation of the polarization resistance can be performed considering this as the real part of the impedance at low frequencies (or from the Bode modulus plot as the interception with the Y axis). This value of polarization resistance is a measure of the protective properties of the oxide coating formed on the steel.

After 30 min of immersion in the solution, no significant differences in terms of Z_R_ at low frequencies, and so in the polarization resistance, could be observed. This indicated that the different corrosion performance recorded in the PDP tests was not related to the characteristics of a passive layer (measured by EIS tests), but to the amount of retained austenite in the samples. In fact, RA, given its low amount of internal defects, reduces the amount of residual stress in the material, making it less susceptible to corrosion. This agreed with the results in the literature [26,27,28].

The data in Table 5 confirmed the results of the qualitative evaluation from the diagrams, in fact the values of R_o_, that is the polarization resistance given by the oxide layer, are similar between the two samples. This allowed us to state that after 30 min of immersion, the volume fraction of RA did not influence the protective properties of the oxide film.

Similarly, after 24 h of immersion, no significant differences between the two specimens are observable in term of polarization resistance, R_o_, both from the qualitative evaluation of the diagrams and from the analysis of the data in Table 5. Moreover, it is possible to observe that a long immersion period led to an increase in terms of R_o_, due to the formation of a thicker passive layer, with semi conductive properties, as demonstrated by Hamadou [43] and Oblonsky [44]. The application of surface-enhanced Raman spectroscopy (SERS) on passive films in borate buffer solutions allowed Oblonsky, in ref. [44], to determine its composition: a mixture of amorphous Fe(OH)_2_, γ-Fe_2_O_3_ and Fe_3_O_4_. It is permissible to think that a passive oxide film with a similar composition is formed on the surface of the steel examined in this work.

## 4. Conclusions

In this work a novel composition of high silicon steel has been studied. It was observed that:

- Austenitizing at 780 °C and austempering at 350 °C produced a dual-phase, ferritic-martensitic microstructure. By increasing the austempering temperature carbide-free bainite was formed, and, as the temperature increased, a coarsening of the bainitic sheaves was observed;
- Increasing the austenitizing temperature from 780 to 850 °C led to an increase in the retained austenite volume fraction, thanks to the increase in bainite amount, which favors carbon partitioning;
- At fixed austenitizing condition (900 °C), the bainite and RA amount increased with the austempering temperature up to 350 °C, whereas at 400 °C no bainite was found due to the absence of bainitic transformation, because 400 °C is above Bs;
- The corrosion resistance of the samples increased with the volume fraction of retained austenite;
- The variation in the corrosion properties, at 30 min of immersion in borate buffer solution, was not linked with variations in the protective properties of the oxide layer (Which did not change from EIS tests), but with the lower amount of residual stresses in the samples with higher amounts of retained austenite;
- The EIS tests, after 24 h of immersion in borate buffer solution, showed that the differences in the RA volume fraction did not produce differences in term of the polarization resistance of the oxide layer even after immersion. The polarization resistance, R_o_, of the sample immersed for 24 h was higher than that of the sample after 30 min of immersion, due to the growth of a thicker passive film.

## Figures and Tables

**Table 1:** High silicon steel chemical composition (wt.%).

Fe	C	Si	Mn	P	S	Cr	Ni	Cu	Mo	Ti	V	Al
Balance	0.430	3.260	2.720	0.010	0.008	0.043	0.074	0.060	0.022	0.001	0.005	0.105

**Table 2:** Scheduled heat treatment.

Heat Treatment Route	Treatment	Austenitizing (°C) (30 min)	Austempering (°C) (30 min)
-	Normalizing	900	-
1	780 + 350	780	350
	830 + 350	830	350
	850 + 350	850	350
2	900 + 300	900	300
	900 + 350	900	350
	900 + 400	900	400

**Table 3:** Results of phase quantification performed by Rietveld analysis.

Heat Treatment	Retained Austenite (vol%)	Bainite/Ferrite (vol%)	Martensite (vol%)
780 (30 min)+ air-cooling (10 °C/s) + 350 (30 min) (40 °C/s) water-cooling	6.2 ± 0.3	45.8 ± 0.3	48.1 ± 0.3
830 (30 min)+ air-cooling (10 °C/s) + 350 (30 min) (40 °C/s) water-cooling	8.7 ± 0.3	36.4 ± 0.3	54.9 ± 0.3
850 (30 min)+ air-cooling (10 °C/s) + 350 (30 min) (40 °C/s) water-cooling	10.7 ± 0.3	40.2 ± 0.3	49.1 ± 0.3
900 (30 min)+ air cooling (10 °C/s) + 300 (30 min) (40 °C/s) water cooling	2.7 ± 0.3	43 ± 0.3	54.3 ± 0.3
900 (30 min)+ air-cooling (10 °C/s) + 350 (30 min) (40 °C/s) water-cooling	5.2 ± 0.3	24.4 ± 0.3	70.4 ± 0.3
900 (30 min)+ air-cooling (10 °C/s) + 400 (30 min) (40 °C/s) water-cooling	1.2 ± 0.3	42.8 ± 0.3	56 ± 0.3

**Table 4:** Values of corrosion potential and corrosion current densities graphically extrapolated form Figure 10a and Figure 11a. Corrosion rates calculated with Equation (1) are also reported in the last two columns.

Heat Treatment	*E_corr_* (V)	*I_corr_* (µA/cm^2^)	Corrosion Rate (g/s × cm^2^)	Corrosion Rate (mm/year)
780 (30 min)+ air-cooling (10 °C/s) + 350 (30 min) (40 °C/s) water-cooling	−0.63	3.5 ± 5%	1.01 × 10^−9^	4.09 × 10^−2^ ± 5%
830 (30 min)+ air-cooling (10 °C/s) + 350 (30 min) (40 °C/s) water-cooling	−0.35	2.5 ± 5%	7.24 × 10^−10^	2.92 × 10^−2^ ± 5%
850 (30 min)+ air-cooling (10 °C/s) + 350 (30 min) (40 °C/s) water-cooling	−0.47	2.3 ± 5%	6.66 × 10^−10^	2.69 × 10^−2^ ± 5%
900 (30 min)+ air-cooling (10 °C/s) + 300 (30 min) (40 °C/s) water-cooling	−0.56	5.0 ± 5%	1.45 × 10^−9^	5.84 × 10^−2^ ± 5%
900 (30 min)+ air-cooling (10 °C/s) + 350 (30 min) (40 °C/s) water-cooling	−0.48	4.6 ± 5%	1.33 × 10^−9^	5.38 × 10^−2^ ± 5%
900 (30 min)+ air-cooling (10 °C/s) + 400 (30 min) (40 °C/s) water-cooling	−0.65	3.2 ± 5%	9.26 × 10^−10^	3.74 × 10^−2^ ± 5%
Normalized 900 (30 min), air-cooling	−0.87	18.0 ± 5%	5.21 × 10^−9^	2.10 × 10^−1^ ± 5%

**Table 5:** Results of the fitting of the experimental data with the equivalent circuit of Figure 14 for the material austempered at 350 °C after austenitizing at 850 °C and in normalized condition.

Equivalent Circuit Values	850 °C 30 min-350 °C 30 min (30 min)	Normalized (30 min)	850 °C 30 min-350 °C 30 min (24 h)	Normalized (24 h)
**R_s_ (Ωcm^2^)**	57.81	54.40	86.38	86.40
**Q_0_ (F × Hz^1-n^)**	1.3 × 10^−4^	1.2 × 10^−4^	2.4 × 10^−4^	2.8 × 10^−4^
**n_O_**	0.74	0.69	0.70	0.68
**R_0_ (Ωcm^2^)**	286.90	290.70	3223	3210
**Χ^2^**	1 × 10^−3^	1 × 10^−3^	1 × 10^−3^	1 × 10^−3^

## Data Availability

The raw/processed data required to reproduce these findings cannot be shared at this time as the data also forms part of an ongoing study.
